# The Obstructive Sleep Apnea Care Ecosystem in the United States: Clinical Pathways and the Evolving Role of Otolaryngology

**DOI:** 10.3390/life16101669

**Published:** 2026-10-07

**Authors:** Xinhang Tu, Alan R. Schwartz, Yichen Wang, Yuting Huang, Yike Li

**Affiliations:** 1 Sleep Disorders Center, Department of Neurology, University of Michigan, Ann Arbor, MI 48109, USA; 2 Department of Otolaryngology-Head and Neck Surgery, Vanderbilt University Medical Center, 1215 21st Ave. S, Medical Center East, Nashville, TN 37232, USA; 3 Department of Otorhinolaryngology, University of Pennsylvania Perelman School of Medicine, Philadelphia, PA 19104, USA; 4 School of Medicine, Universidad Peruana Cayetano Heredia, Lima 15102, Peru; 5 Division of Gastroenterology and Hepatology, Mayo Clinic, Jacksonville, FL 32224, USA

**Keywords:** obstructive sleep apnea, otolaryngology, polysomnography, positive airway pressure, drug-induced sleep endoscopy, neurostimulation, upper-airway phenotyping, weight management

## Abstract

Obstructive sleep apnea (OSA) is a common, heterogeneous disorder managed through a multidisciplinary pathway that connects recognition, physiologic testing, treatment selection, specialty referral, insurance coverage, and longitudinal reassessment. This narrative review provides a clinically oriented, system-level overview of the adult OSA care ecosystem in the United States for an international clinical audience, with particular emphasis on the evolving role of otolaryngology. We describe the organization and complementary functions of sleep physicians, otolaryngologists, dentists, and allied health professionals. We also outline the roles of polysomnography and home sleep apnea testing, along with major therapeutic pathways including positive airway pressure, oral appliance therapy, weight management, upper-airway surgery, and neurostimulation. Otolaryngology provides an important interface between physiologic diagnosis and airway-specific treatment by integrating anatomic evaluation, sleep-state airway phenotyping, intervention selection, and post-treatment reassessment. Recent advances in neurostimulation, pharmacologic weight management, wearable and home-based monitoring, airflow analysis, and artificial intelligence are expanding opportunities for accessible and phenotype-informed care. Important challenges include underdiagnosis, treatment attrition, fragmented transitions, variable coverage, and reliance on the apnea–hypopnea index. Although health-care structures differ internationally, multidisciplinary coordination, individualized treatment selection, and longitudinal reassessment provide broadly applicable principles for developing patient-centered OSA programs.

## 1. Introduction

Obstructive sleep apnea (OSA) is a common sleep-related breathing disorder whose heterogeneous presentation and multisystem consequences call for coordinated care [1,2]. In the United States, diagnosis and treatment are delivered through an established multidisciplinary care infrastructure linking sleep medicine, primary and specialty care, technical and allied professionals, and a broad range of medical, behavioral, device-based, and surgical therapies. This narrative review provides a practical map of that infrastructure for an international clinical audience, tracing how professionals are trained and organized, how adults with OSA move from recognition to testing and treatment, and how referral and coverage frameworks shape care. Rather than focusing on a single diagnostic or therapeutic modality, the review integrates the clinical, professional, and health-system components that collectively shape contemporary OSA care in the United States, with particular attention to the evolving role of otolaryngology in translating physiologic diagnosis into airway-specific, phenotype-informed intervention. Because the review was designed as a clinically oriented overview of the U.S. OSA care system rather than to address a prespecified research question, a formal systematic search strategy and predefined inclusion criteria were not used. References were selected to support the major components of this clinical framework, with emphasis on relevant professional guidelines and consensus statements, landmark studies, and contemporary evidence informing current practice. Together, these pathways illustrate a broader model of sleep care in which multidisciplinary expertise is organized around the needs of the individual patient.

## 2. Why OSA Matters

OSA is common yet underrecognized. Population studies show that its prevalence increases with age and adiposity, although reported estimates vary according to the population studied, including age, sex, obesity burden, and comorbidities, as well as the diagnostic method and criteria used to define respiratory events. In the United States, moderate-to-severe OSA (apnea–hypopnea index [AHI] ≥ 15 events/h) affects approximately 10–17% of men and 3–9% of women aged 30–70 years [3,4]. Globally, OSA defined by AHI ≥ 5 events/h has been estimated in 9–38% of adults, and a substantial proportion of moderate-to-severe cases remain undiagnosed [4,5]. More recent estimates suggest that overall OSA prevalence may be substantially higher depending on the population and diagnostic criteria applied [6].

The clinical burden of OSA extends across symptoms, organ systems, and society. OSA contributes to excessive daytime sleepiness, neurocognitive deficits, mood disturbance, reduced work performance, and lower quality of life [7]. Observational studies have also linked untreated OSA with hypertension, arrhythmias, heart failure, stroke, type 2 diabetes, and motor-vehicle crashes, with higher all-cause mortality reported particularly in severe OSA [1,8]. These associations vary in strength across outcomes and patient populations, and observational data do not by themselves establish causality. Clinical severity therefore cannot be defined by AHI alone; symptom burden, degree and pattern of hypoxemia, comorbidities, and relevant anatomic and physiologic characteristics may also inform risk assessment and treatment selection. Undiagnosed OSA further imposes substantial costs through health-care use, lost productivity, accidents, and workplace injury [9].

Patients may therefore present with sleep-related symptoms, cardiometabolic conditions, perioperative risk concerns, or upper-airway pathology. This clinical diversity places OSA across the boundaries of multiple specialties and creates a need for coordinated multidisciplinary pathways spanning case identification, diagnostic evaluation, individualized treatment selection, and longitudinal follow-up.

## 3. Training and Organization of OSA Care

The clinical breadth of OSA is reflected in the organization of sleep care. In the United States, there is no single uniform model of OSA care, and organizational structures vary across academic centers, community practices, integrated health systems, and regions with more limited access to specialized services. In many settings, the sleep program serves as the longitudinal coordinating hub, while other disciplines contribute at defined points according to the patient’s presentation, comorbidities, treatment needs, and airway phenotype. The framework described below therefore represents common components of U.S. practice rather than a universally applied care model.

### 3.1. Physicians

Sleep medicine is an American Board of Medical Specialties (ABMS)-recognized subspecialty pursued after primary residency training, most commonly in internal medicine, neurology, psychiatry, and pediatrics. Physicians trained in family medicine, otolaryngology–head and neck surgery, and anesthesiology may also enter the field [10]. Training occurs through a one-year Accreditation Council for Graduate Medical Education (ACGME)-accredited fellowship, followed by subspecialty certification through the pathway applicable to the physician’s primary specialty. This cross-board structure establishes shared competence in sleep physiology, diagnostic testing, and longitudinal management while preserving the complementary perspective of each physician’s original discipline [11,12].

In clinical practice, sleep medicine physicians integrate the clinical history, comorbidities, examination findings, and objective sleep testing; establish the diagnosis; initiate and monitor therapy; assess longitudinal treatment response; and coordinate referral to other specialists when appropriate. Their role therefore extends beyond interpretation of sleep studies with PAP initiation to longitudinal management of OSA as a chronic disorder.

### 3.2. Related Specialties

Other disciplines contribute distinct expertise across the diagnostic and therapeutic pathway. Otolaryngologists evaluate structural and inflammatory contributors to upper-airway obstruction, characterize dynamic collapse, address nasal and upper-airway factors that may interfere with other therapies, and evaluate selected patients for airway-directed surgery and neurostimulation, particularly when anatomy contributes to obstruction or positive airway pressure (PAP) is poorly tolerated or ineffective [13,14]. Qualified dentists deliver custom oral appliance therapy (OAT) in collaboration with sleep clinicians [15]. Behavioral sleep specialists address comorbid insomnia and sleep-related behavioral barriers [16], while primary care, cardiology, pulmonology, neurology, endocrinology, bariatric medicine, nutrition services, and perioperative teams identify at-risk patients and co-manage associated disease [17,18].

Training and credentialing for these disciplines are governed by their respective specialty boards and societies [10], while integration with sleep clinics is achieved through institutional protocols, privileging, shared documentation, defined referral pathways, and coordinated outcome assessment. This structure allows behavioral, dental, anatomic, and cardiometabolic expertise to be incorporated when clinically relevant without duplicating the core diagnostic and longitudinal functions of the sleep program.

### 3.3. Advanced Practice, Technical, and Clinical Support Workforce

Advanced practice providers (APPs), including nurse practitioners and physician assistants, expand access and continuity of care by conducting intake and follow-up visits, reviewing PAP data, troubleshooting common issues, and providing patient education. Their specific responsibilities may vary according to state scope-of-practice requirements and institutional policies. Sleep technologists and respiratory therapists provide the technical foundation for diagnostic testing and PAP titration, with professional credentialing pathways through organizations such as the Board of Registered Polysomnographic Technologists (BRPT) and the National Board for Respiratory Care (NBRC).

These professionals enter sleep care through different training and credentialing pathways. Many institutions supplement this preparation with structured onboarding and American Academy of Sleep Medicine (AASM) educational resources, including sleep-medicine training for APPs and the Accredited Sleep Technologist Education Program (ASTEP) for technologists [19,20]. Nurses, medical assistants, PAP educators, schedulers, and durable-medical-equipment personnel further connect clinical recommendations with equipment delivery, education, early troubleshooting, and follow-up. Depending on the care model and payer, durable medical equipment suppliers may also coordinate PAP setup and mask fitting, replacement supplies, transmission of device-use data, and documentation needed for insurance authorization or continued coverage. Together, this workforce helps translate a diagnostic or therapeutic plan into care that can be implemented and sustained, supporting adherence, early resolution of treatment-related problems, and continuity of care.

### 3.4. Program Standards

Several related but distinct concepts shape the organization of sleep care in the United States. Accreditation evaluates whether a health care program or practice meets defined organizational and quality standards; professional certification documents that an individual has met requirements within a defined area of expertise; licensure provides legal authorization to practice a profession within a state; and insurance requirements determine whether particular tests, devices, or treatments qualify for coverage or reimbursement. These functions may interact operationally but are not interchangeable.

AASM accreditation provides a professional and organizational framework for sleep-care delivery across sleep clinics, in-lab polysomnography (PSG) testing, home sleep apnea testing (HSAT) services, and applicable durable-medical-equipment services [21]. Accreditation sets standards for medical leadership, staff qualifications and competencies, testing and scoring procedures, quality assurance, and operational policies intended to support consistent and high-quality practice. Multiple service types may be organized under shared governance, which can facilitate coordination and consistency across sites and stages of care, although accreditation does not imply uniform practice patterns or clinical outcomes across programs. Selected U.S. professional, accreditation, credentialing, and regulatory organizations relevant to OSA care are summarized in Table 1.

## 4. Diagnostic Evaluation and Referral

### 4.1. Patient Entry and Clinical Triage

**Figure 1:**
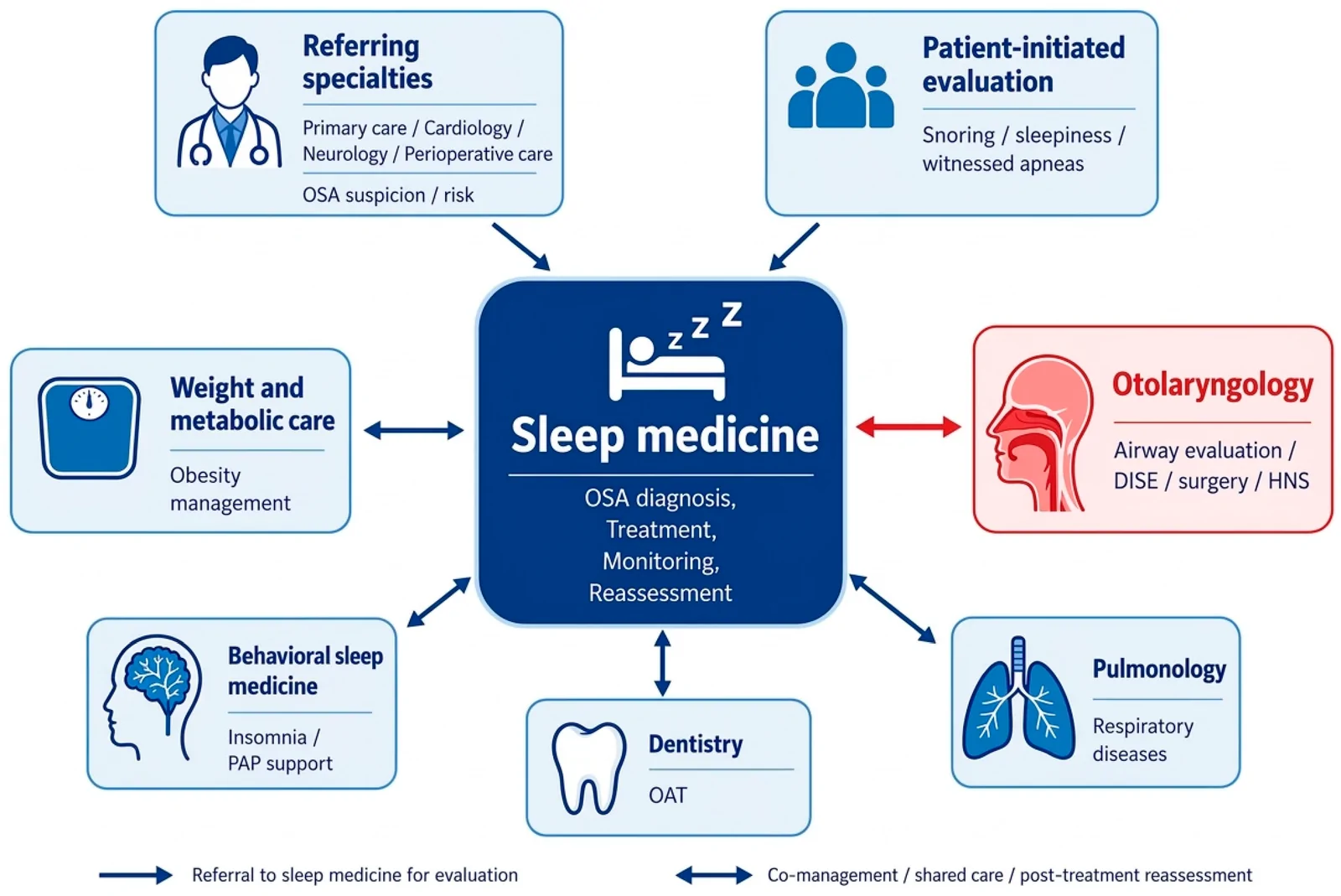
**Multidisciplinary Care Pathways for Adult OSA in the United States**. Sleep medicine commonly serves as the central coordinating specialty for OSA diagnosis, treatment, monitoring, and reassessment. Patients may enter the pathway through referral from primary or specialty care or through patient-initiated evaluation. Depending on treatment needs and comorbidities, care may involve dentistry, otolaryngology, pulmonology, behavioral sleep medicine, and weight or metabolic services. Single arrows indicate referral to sleep medicine for evaluation; bidirectional arrows indicate co-management, shared care, or post-treatment reassessment. Red highlighting emphasizes the airway-focused role of otolaryngology within the multidisciplinary OSA care pathway. Abbreviations: DISE, drug-induced sleep endoscopy; HNS, hypoglossal nerve stimulation; OAT, oral appliance therapy; OSA, obstructive sleep apnea; PAP, positive airway pressure.

In the United States, patients may enter the OSA pathway through primary care or a range of specialty settings, depending on symptoms, referral patterns, health-system structure, and access to specialty care. Evaluation may be prompted by snoring, witnessed apneas, excessive daytime sleepiness, resistant hypertension, or cardiometabolic comorbidities. Cardiology, neurology, bariatric and weight-management, perioperative, dental, and otolaryngology settings also generate frequent referrals, and patient-initiated evaluation is increasingly common (Figure 1).

Brief questionnaires support triage, although they do not establish the diagnosis. STOP-BANG is sensitive for moderate-to-severe OSA and is widely used in perioperative screening [22], while the Berlin Questionnaire provides another approach to risk stratification [23]. The Epworth Sleepiness Scale (ESS; range 0–24) quantifies subjective sleepiness and can help track symptomatic response over time [24], but it should not be interpreted as a diagnostic or stand-alone screening test for OSA. These tools are best interpreted within a comprehensive clinical evaluation that considers symptoms, comorbidities, competing sleep disorders, and the purpose of testing.

### 4.2. Physiologic Testing

Diagnosis of OSA in adults requires either PSG or technically adequate HSAT performed in the context of a comprehensive sleep evaluation [25,26] (Table 2). HSAT is appropriate for uncomplicated adults with suspected moderate-to-severe OSA. PSG is preferred when significant cardiorespiratory disease, neuromuscular weakness, suspected hypoventilation, chronic opioid use, prior stroke, severe insomnia, or another sleep disorder may complicate interpretation. A negative, inconclusive, or technically inadequate HSAT is generally followed by PSG when clinical suspicion remains high. A split-night study may combine diagnosis and PAP titration when substantial OSA is identified early in the study [26].

Traditional Type I-IV classification (Table 3) and the signal-based SCOPER framework (Sleep, Cardiovascular, Oximetry, Position, Effort and Respiratory) describe the testing setting and physiologic channels available in a study [27,28]. Although these classifications remain useful, contemporary home sleep testing devices vary considerably in sensor configuration and technology, and payer-specific technical requirements may not correspond exactly to the traditional Type I-IV framework. Their practical purpose is to clarify what a test can and cannot measure. Test selection is best guided by patient complexity and the information required for the next decision.

### 4.3. Study Interpretation

Interpretation of a sleep study addresses two related questions: the severity and physiologic burden of sleep-disordered breathing, and the predominant type of respiratory disturbance. PSG commonly reports the AHI and respiratory disturbance index (RDI), with the latter incorporating respiratory effort-related arousals where applicable. HSAT generally reports a respiratory event index (REI) because sleep time and arousals may not be directly measured [25,26,29]. Thus, AHI, RDI, and REI are related but not interchangeable: AHI generally reflects apneas and hypopneas per hour of sleep, RDI may additionally include respiratory effort-related arousals, and REI generally expresses respiratory events relative to monitoring or recording time when EEG-defined sleep time is unavailable. In adults, OSA is commonly categorized by event frequency as mild (5 to <15 events/h), moderate (15 to <30 events/h), or severe (≥30 events/h), although these categories should be interpreted within the broader clinical context. Reports may also include the oxygen-desaturation index, oxygen-saturation nadir, time below a specified threshold, sleep-stage and positional distributions, and the relative burden of obstructive, central, and mixed events. In appropriate clinical settings, testing may also identify patterns suggestive of sleep-related hypoventilation or underlying cardiopulmonary disease. AASM hypopnea scoring permits ≥ 3% desaturation and/or arousal, whereas Medicare uses a ≥4% desaturation criterion for coverage determinations [29,30,31]. These scoring differences can alter the calculated AHI and, in some patients, the assigned severity category or eligibility for treatment under specific payer policies. Many laboratories in the United States therefore report both values when needed for clinical and payer purposes.

AHI remains clinically useful, but event counts alone do not fully describe disease burden. Oxygenation, symptoms, comorbidities, and in selected settings, measures such as hypoxic burden can add context for risk assessment and follow-up [32]. Hypoxic burden remains a promising complementary measure rather than a routine clinical standard and has not replaced AHI within current regulatory or reimbursement frameworks. Consumer sleep technologies may support awareness and longitudinal observation, but device performance and validation vary substantially [33]. These technologies are therefore best positioned as complements to clinical evaluation and validated diagnostic testing.

### 4.4. From Results to Referral

Following diagnosis, PAP commonly anchors initial therapy for clinically significant OSA, but treatment selection should be individualized through shared decision-making and incorporate disease severity, symptoms, oxygen desaturation, relevant anatomy, comorbidities, patient preference, prior treatment experience and treatment feasibility [34]. Patients who prefer OAT or cannot use PAP may be referred to a qualified dentist [15,35]. Obesity may prompt coordinated weight-management, endocrinology, or bariatric care [13,17], while complex ventilatory findings may require further sleep or pulmonary evaluation.

Otolaryngology becomes particularly relevant when the unresolved question concerns the upper airway, including clinically important nasal obstruction, suspected surgically modifiable anatomic obstruction, upper-airway factors contributing to PAP intolerance, or evaluation for upper-airway surgery or hypoglossal nerve stimulation (HNS) [13,14,36,37]. At this point, evaluation moves from confirming OSA to determining where and how the airway collapses and which features may be amenable to intervention. Testing, referral, and treatment selection thus remain parts of one connected and individualized pathway.

## 5. Management Options

OSA is a chronic disorder requiring longitudinal, individualized and outcomes-oriented care. Treatment is informed by disease burden, symptoms, comorbidities, anatomy and physiology, prior treatment experience, and patient preference. Available therapies form a portfolio that can be selected, sequenced, or combined according to the clinical context. Across modalities, effectiveness is assessed through both objective control and patient-reported benefit.

### 5.1. Positive Airway Pressure

PAP remains the first-line treatment for many adults with clinically significant OSA [34]. By pneumatically stabilizing the upper airway, it reduces obstructive events and improves sleepiness, quality of life, and blood pressure in selected patients. Treatment may be initiated using home-based automatic PAP in uncomplicated OSA or laboratory titration in more complex cases. Longitudinal management includes review of treatment adherence, residual events, mask leak, comfort, and symptoms. Clinical effectiveness depends not only on physiologic control while PAP is used but also on actual patterns of use, tolerability, and symptomatic response. Payer-defined adherence thresholds are primarily coverage criteria and do not necessarily represent an individualized threshold for clinical benefit [34,38].

When conventional PAP is ineffective or poorly tolerated, evaluation of mask-interface, nasal, pressure-related, behavioral, and support-related barriers can identify correctable problems and clarify whether transition to another therapeutic pathway is appropriate. Alternative PAP modes, such as bilevel PAP, may be appropriate for selected patients according to pressure requirements and coexisting ventilatory disorders [34].

### 5.2. Non-PAP and Metabolic Pathways

Custom, titratable mandibular advancement devices provide an evidence-based alternative, particularly for patients with mild-to-moderate OSA and for those who prefer an alternative to PAP or cannot use it effectively [15]. They are fitted and monitored by qualified dentists, with follow-up sleep testing used to confirm efficacy. Long-term use may be associated with progressive dental and occlusal effects, including changes in overjet, overbite, and tooth position, as well as discomfort or temporomandibular symptoms in some patients. Periodic dental assessment is therefore important to monitor treatment tolerance and occlusal changes over time [15,35]. Positional therapy, delivered through physical positioning strategies or vibrotactile wearable devices, may benefit patients whose disease is substantially worse during supine sleep, although long-term effectiveness depends on sustained use [39].

Weight management is a cross-cutting component of care when obesity contributes to OSA. Lifestyle intervention and bariatric surgery remain important options, while tirzepatide (Zepbound) has introduced a pharmacologic pathway for adults with obesity and moderate-to-severe OSA [17,40,41,42]. Obesity management medications should be used in conjunction with reduced-calorie diet and increased physical activity, and they should be viewed as part of an individualized OSA treatment strategy rather than a replacement for OSA-specific therapy. Pharmacologic weight management is generally delivered within a broader program that includes nutritional and behavioral support. Longitudinal monitoring of body weight, treatment tolerance, and sleep-disordered breathing can guide subsequent adjustment of both metabolic and airway-directed therapies. Because improvement in OSA after weight loss varies between individuals, clinical and objective sleep reassessment should precede de-escalation or discontinuation of established OSA therapy.

### 5.3. Airway-Directed Therapy

Upper-airway surgery addresses anatomic contributors and is typically considered when PAP is not feasible or when assessment identifies a modifiable anatomic target. Patient selection should consider OSA severity, symptoms, upper-airway anatomy, comorbidities, prior treatment experience, anticipated procedural benefit, and patient preference. Procedures range from nasal surgery—which often primarily improves nasal breathing and facilitates PAP or OAT tolerance rather than directly normalizing OSA—to palatal or multilevel soft-tissue surgery and skeletal approaches such as maxillomandibular advancement. Outcomes are most meaningful when the intended anatomic target and therapeutic goal are clearly defined before surgery and reassessed afterward [13,43,44,45].

HNS is an established device-based option for selected adults with moderate-to-severe OSA who cannot use PAP effectively. By stimulating tongue-protruding muscles, HNS promotes anterior displacement of the tongue and helps maintain upper-airway patency during sleep [37,46]. Treatment involves coordinated selection, including evaluation of airway-collapse patterns where applicable, followed by implantation, programming, and follow-up testing [36,47]. Eligibility depends on clinical severity, prior treatment response, anatomy, and device-specific and payer-specific criteria; therefore, individual age, AHI, body-mass-index, and airway thresholds are not universal across all HNS systems.

### 5.4. Emerging and Investigational Therapies

Several newer approaches illustrate the expanding therapeutic landscape but differ substantially in regulatory status and evidentiary maturity. Emerging pressure-delivery algorithms, including Kairos PAP (KPAP), are being investigated for their potential to improve comfort while maintaining event control [48]. Current evidence for KPAP is limited to two randomized trials reported in a single publication, and further independent studies are needed to establish its longer-term effectiveness, adherence, and role in clinical practice.

Daytime neuromuscular tongue stimulation (e.g., eXciteOSA) is an FDA-authorized option for selected adults with mild OSA [49], whereas myofunctional therapy is generally considered an adjunctive behavioral approach [50]. Ansa cervicalis stimulation remains investigational and is being studied as a potential means of modifying additional components of pharyngeal airway collapse [51,52,53,54]. AD109, an oral neuromuscular pharmacologic therapy, has completed phase 3 evaluation but remains investigational while undergoing regulatory review [55,56]. These therapies should therefore be interpreted according to their individual evidence base, regulatory status, and target population rather than as equivalent alternatives to established treatments.

## 6. The Role of Otolaryngology in OSA Care

Otolaryngology provides the clinical interface between a physiologic diagnosis of OSA and an airway-specific, phenotype-informed treatment strategy. Otolaryngologists commonly enter the care pathway when anatomic obstruction is suspected, PAP is poorly tolerated or provides inadequate control, OAT, surgery, or neurostimulation is being considered, or the response to prior treatment is incomplete. Their contribution lies in identifying modifiable components of upper-airway obstruction and placing airway-directed intervention within the overall individualized treatment plan.

### 6.1. Airway Evaluation and Phenotyping

Evaluation begins with the clinical history and awake examination of nasal patency, mucosal or inflammatory disease, tonsillar and palatal anatomy, tongue-base crowding, and craniofacial structure. Because OSA is expressed during sleep, static findings do not fully represent dynamic obstruction. Drug-induced sleep endoscopy (DISE) can characterize the level, direction, and degree of collapse in selected patients [57,58], often using the VOTE (Velum, Oropharynx, Tongue base, Epiglottis) framework [59].

DISE has an established role in aspects of HNS selection and may also inform consideration of mandibular advancement, positional strategies, and site-directed surgery [14,36,47,58]. In selected research and clinical settings, DISE has also been combined with PAP titration and other controlled maneuvers to examine changes in airway behavior [52,53,60,61]. DISE also has recognized limitations, including variability in interpretation and potential influence of sedation depth and technique on observed collapse patterns. Standardized protocols and reporting frameworks can improve consistency, but findings should not be interpreted in isolation [57]. DISE findings are most informative when integrated with objective sleep testing, awake anatomy, previous treatment experience, and patient goals.

Importantly, airway anatomy and dynamic collapse represent only one dimension of OSA. Broader characterization may also incorporate clinical phenotypes, such as symptom burden, as well as physiologic endotypes, including ventilatory-control instability, arousal threshold, upper-airway muscle responsiveness, and anatomic collapsibility. Otolaryngologic assessment therefore contributes an airway-focused component to a broader multidimensional characterization of OSA.

### 6.2. From Phenotype to Intervention

Otolaryngologist-directed treatment is guided by the anatomic or functional target and the intended clinical outcome. Nasal surgery may improve breathing or facilitate PAP use [45]; tonsillar, palatal, tongue-base, lateral-wall, or skeletal procedures may address dominant sites of obstruction [43,44]; and HNS provides a neuromuscular option for selected patterns of airway collapse [37]. An otolaryngology consultation may also lead to PAP optimization, OAT, weight management, additional evaluation, or a combination strategy rather than an operation. Within a shared decision-making framework, treatment selection incorporates expected efficacy, invasiveness, reversibility, recovery, treatment burden, and patient priorities [13].

### 6.3. Coordination and Outcome Verification

The role of otolaryngology continues through the perioperative and post-treatment course. Preoperative planning integrates disease severity, comorbidities, airway findings, PAP experience, and perioperative risk [13,62,63]. Post-treatment assessment incorporates symptoms and patient-reported outcomes together with objective testing when appropriate [13,15,64]. Improvement in snoring alone does not establish adequate control of OSA. Residual disease may prompt device reprogramming or the addition of complementary strategies such as positional therapy, weight management, an oral appliance, or another airway-directed intervention [15,64]. By connecting diagnosis, airway-focused phenotyping, intervention, and reassessment, otolaryngology helps close the loop within multidisciplinary OSA care.

## 7. Insurance and Coverage

In the United States, clinical pathways are also shaped by insurance definitions and documentation requirements. Coverage policies are not uniform: Medicare and private insurers may apply different diagnostic, documentation, prior-authorization, adherence, and treatment-eligibility criteria. A practical example is the difference between AASM and Medicare hypopnea criteria: Medicare’s requirement for a ≥4% oxygen desaturation may produce a lower coverage-based AHI or REI than an index calculated using the AASM criterion of ≥3% desaturation and/or arousal [29,30,31]. Many sleep laboratories therefore report indices using both definitions when needed for clinical interpretation and payer documentation. Selection of a diagnostic test may consequently reflect both the clinical question and the requirements of the anticipated treatment pathway.

Coverage for newer otolaryngology-directed therapies remains plan-specific. For HNS, payers commonly require a qualifying sleep study, predominantly obstructive rather than central respiratory events, documentation of PAP failure or intolerance, appropriate upper-airway anatomy and DISE findings, and additional eligibility criteria. Some payers retain AHI or body mass index thresholds that are narrower than current regulatory labeling [65,66]. Prior-authorization and documentation requirements can delay access to treatment even when a therapy is clinically appropriate. Complete documentation of the sleep study, PAP history, airway evaluation, treatment rationale, and planned follow-up may help reduce avoidable delays and facilitate the transition to an airway-directed pathway.

Coverage for weight-management interventions is also plan-specific and may affect treatment access and sequencing. Across treatment pathways, coverage determines access under a particular policy, whereas individualized clinical judgment remains grounded in disease burden, anatomy, treatment feasibility, and patient preferences. Accordingly, coverage criteria should be distinguished from clinical eligibility and individualized treatment selection.

## 8. Challenges and Future Directions

Access and variation in care remain important challenges within the U.S. OSA care system. Despite its established multidisciplinary infrastructure, OSA care remains limited by underdiagnosis, barriers to access, treatment attrition, and fragmentation between services [6,67,68,69,70,71]. Availability of sleep specialists, diagnostic testing, multidisciplinary expertise, and advanced therapies varies across regions and health systems, contributing to unequal access to evaluation and treatment. Early education, troubleshooting, telemonitoring, and streamlined referral pathways may help patients initiate and sustain treatment, although institutional capacity varies and important inequities persist [69,72,73]. Continued efforts will be necessary to improve both access to initial evaluation and transitions from diagnosis to treatment, from first-line therapy to an effective alternative, and from intervention to longitudinal reassessment.

A central scientific challenge is the reliance on AHI as the principal measure of OSA severity. AHI remains simple, familiar, and clinically useful, but correlates imperfectly with symptoms, cardiometabolic outcomes, and treatment response [2,32,74,75]. A more comprehensive description of disease may incorporate oxygenation and hypoxic burden, positional and sleep-stage dependence, comorbidities, symptoms, and patient-reported outcomes alongside event frequency [2,24,32,75,76,77,78]. At present, however, no alternative metric has achieved broad consensus as a replacement for AHI or comparable recognition within regulatory and reimbursement frameworks. Observable phenotypes may also provide information about underlying pathophysiologic traits, or endotypes, including anatomic collapsibility, ventilatory-control instability, arousal threshold, and upper-airway muscle responsiveness [2]. The greatest clinical value is likely to come from reproducible and actionable features that refine risk assessment, treatment selection, or evaluation of response.

Digital and computational tools offer complementary opportunities to support this transition. Telemedicine and remote monitoring can extend selected components of testing and follow-up beyond conventional clinical visits [69,72,73]. Wearables and home-based sensors may facilitate repeated measurement [33,79], while analysis of routine PSG/HSAT signals, including breath-by-breath airflow morphology [80,81], may reveal physiologic patterns not represented by AHI alone. Artificial intelligence may assist signal scoring, phenotyping, risk assessment, and clinical prioritization [82,83]. Many of these applications remain under validation and should be viewed as potential complements rather than replacements for established clinical evaluation. Translation into patient care will depend on external validation, interpretability, generalizability, reproducibility across populations and devices, and evidence that the resulting information improves clinical decisions or outcomes.

For otolaryngology, a particularly relevant opportunity lies in linking physiologic signals with the structure and dynamic behavior of the upper airway. Integration of sleep-study signals, airflow characteristics, and endoscopic or anatomic findings may eventually help identify patients most likely to benefit from direct airway evaluation or a specific intervention. This approach could extend airway phenotyping beyond a single procedure and provide clinically accessible surrogates for treatment-relevant patterns of collapse [81,84,85].

Treatment development is similarly moving toward target-based selection. Metabolic therapy [17,41,86], investigational pressure-delivery approaches [48], emerging upper-airway pharmacotherapy [56], surgery [13,43], and neurostimulation [37,51,53,61] act on different components of OSA and may increasingly be sequenced or combined according to the dominant and residual treatment targets. These approaches differ substantially in evidentiary maturity and regulatory status, as discussed above, and their roles will require continued comparative and longitudinal evaluation. The emerging goal is an effective and sustainable treatment strategy that minimizes treatment burden for the individual patient. Together, these developments point toward an adaptive care pathway that integrates equitable access, clinically meaningful phenotypes and physiologic endotypes, targeted intervention, and objective reassessment.

## 9. Conclusions

Modern OSA care in the United States operates through a multidisciplinary care framework that combines professional training, diagnostic testing, multiple therapeutic modalities, and longitudinal follow-up within diverse institutional and payer structures. This framework integrates primary and specialty care with sleep medicine, dentistry, behavioral and metabolic services, technical support, and longitudinal outcome assessment to address the heterogeneous clinical manifestations and treatment needs of OSA. Care delivery varies across settings, but the interaction among these disciplines illustrates how different components of care can be coordinated around individual patient needs. Within this system, otolaryngology provides a distinctive bridge between the physiologic diagnosis of OSA and an airway-specific treatment strategy, integrating anatomy, sleep-state airway behavior, previous treatment experience, patient goals, and objective sleep data to guide intervention and verify benefit.

The field is advancing toward broader measures of disease burden, more accessible longitudinal monitoring, and treatment strategies increasingly informed by anatomy, physiology, and patient priorities. Many emerging diagnostic metrics, digital technologies, and therapeutic approaches will require further validation before their roles in routine care are established. Although workforce, referral, and coverage structures differ across countries, the central principles are broadly shared: efficient recognition, interpretation in clinical context, treatment matched to modifiable targets, and objective and patient-centered reassessment. Sustained international collaboration and open exchange of methods, data, technologies, and clinical outcomes will allow insights from the United States and other health-care systems to inform one another. Such cooperation can strengthen locally tailored models of care while collectively advancing OSA management and the broader field of sleep medicine.

## Figures and Tables

**Table 1:** Selected U.S. Organizations and Training or Credentialing Programs Relevant to OSA Care.

Organization or Program	Primary Role in OSA Care
American Academy of Sleep Medicine (AASM)	Clinical practice guidance, sleep-center accreditation, professional education, and sleep-medicine standards
Accredited Sleep Technologist Education Program (ASTEP)	AASM-developed education and training pathway for sleep technologists and other sleep-care personnel
Accreditation Council for Graduate Medical Education (ACGME)	Accreditation of sleep-medicine fellowship training programs
American Board of Medical Specialties (ABMS) and member boards	Physician subspecialty certification in sleep medicine
American Academy of Dental Sleep Medicine (AADSM)	Professional guidance and education related to oral appliance therapy
American Academy of Otolaryngology–Head and Neck Surgery (AAO-HNS)	Professional guidance and education related to otolaryngologic and airway-directed care
Board of Registered Polysomnographic Technologists (BRPT)	Credentialing of sleep technologists
National Board for Respiratory Care (NBRC)	Credentialing of respiratory therapists, including sleep-related credentials
Centers for Medicare & Medicaid Services (CMS)	Federal coverage and reimbursement requirements for eligible beneficiaries

**Table 2:** **Comparison Between In-Laboratory Polysomnography and Home Sleep Apnea Testing.** Abbreviations: ECG, electrocardiography; EEG, electroencephalography; EMG, electromyography; EOG, electrooculography; HSAT, home sleep apnea testing; OSA, obstructive sleep apnea; PLMS, periodic limb movements of sleep; PSG, polysomnography.

Feature	In-Lab Polysomnography (PSG)	Home Sleep Apnea Testing (HSAT)
**Location**	Sleep laboratory	Patient’s home
**Parameters Measured**	Multiple (EEG, EOG, EMG, ECG, airflow, respiratory effort, SpO_2_, etc.)	Limited cardiorespiratory signals; typically no EEG (airflow, respiratory effort, SpO_2_)
**Sleep Staging**	Yes (full sleep architecture assessed)	Usually no
**Diagnostic Scope**	Broad evaluation of sleep disorders requiring PSG	Primarily OSA in appropriately selected patients
**Supervision**	Yes (technician-monitored)	No (unattended; some devices allow remote monitoring)
**Diagnostic Performance**	Comprehensive physiologic assessment with direct sleep staging	Adequate for OSA diagnosis in appropriately selected patients; performance varies by device and patient population
**Cost**	Higher	Lower
**Availability**	Limited by laboratory capacity	Often more available
**Comfort/Convenience**	Less comfortable (wires, sensors, away from home)	More comfortable (at home, fewer wires/equipment)
**Insurance Coverage**	Varies by indication and payer	Varies by indication, device, and payer
**Contraindications**	Few (e.g., severe illness may preclude lab visit)	Not recommended for comorbid conditions or complex sleep disorders

**Table 3:** **Traditional Classification of Sleep-Study Types**. The Type I–IV system describes studies according to setting, supervision, and recording capability. Contemporary HSAT devices vary in sensor configuration, and payer-specific technical requirements may differ from this traditional classification. Abbreviations: ECG, electrocardiography; EEG, electroencephalography; EMG, electromyography; EOG, electrooculography; OSA, obstructive sleep apnea; PAP, positive airway pressure; PSG, polysomnography.

Type	Location	Channels Recorded	Tech Supervision	Main Uses
I	Lab	Full PSG (≥7 channels, including EEG, EOG, EMG)	Yes	Comprehensive PSG evaluation
II	Home/Lab	Full PSG	No	Unattended PSG
III	Home	≥4 channels: respiratory signals, oxygen saturation, cardiac signal	No	OSA diagnosis
IV	Home	1–2 (typically oxygen saturation and heart rate)	No	Limited-channel OSA evaluation

Note: Medicare-covered Type IV testing requires at least three channels, including airflow.

## Data Availability

No new data were created or analyzed in this study. Data sharing is not applicable to this article.
